# Transarterial Radioembolization (TARE) Complicated by a Mycotic Aneurysm and Bleeding: A Case Presentation and Literature Review

**DOI:** 10.7759/cureus.98484

**Published:** 2025-12-04

**Authors:** Eddie H Hu

**Affiliations:** 1 Department of Hematology and Oncology, University of California, Los Angeles (UCLA) Health, Alhambra, USA

**Keywords:** bleed, hepatocellular carcinoma, liver abscess, mycotic aneurysm, transarterial radioambolization

## Abstract

Hepatocellular carcinoma (HCC) is a primary cancer of the liver, typically developing in the setting of chronic liver disease, especially in patients with cirrhosis related to chronic hepatitis B or C virus infections, nonalcoholic steatohepatitis (NASH), or chronic alcohol abuse. HCC is the third leading cause of cancer-related deaths in the world and one of the fastest-growing causes of cancer death in the United States. The reason for this poor prognosis of most HCC patients is the advanced stage at diagnosis. HCC patients are frequently asymptomatic at early stages, and screening even for high-risk individuals is typically lacking. Our case is illustrative of this.

A male patient had chronic liver disease from NASH, but was never screened for liver cancer. At presentation, he had a tumor measuring over 6 cm, which was considered unresectable due to its size and the presence of underlying cirrhosis. For patients with limited intrahepatic tumor burden with unresectable tumors, a locoregional approach is normally recommended, as opposed to systemic treatment. The choice of local regional therapies includes local thermal ablation, transarterial chemoembolization (TACE), transarterial radioembolization (TARE), hepatic artery infusional chemotherapy (HAIC), and stereotactic body radiation (SBRT). Both thermal ablation and SBRT work better for smaller tumors that measure less than 3 cm. HAIC is limited to centers with technical expertise. TACE and TARE are the primary options for this condition. Meta-analyses comparing TACE versus TARE suggest TARE provides significantly longer time to progression compared with TACE, with less toxicity. It also entails fewer treatments but at a higher cost.

Our patient opted for TARE, which resulted in good tumor regression but was complicated by the development of a liver abscess in the treated area. Liver abscess is a rare complication of TARE. Following embolization, the tumor tissue dies, and in rare cases, this area of necrosis becomes infected, forming an abscess. This infection may be due to bacteria from a compromised biliary tract or the bloodstream. An extremely rare secondary complication of the liver abscess is the development of a mycotic aneurysm in the branch of the hepatic artery surrounded by the liver abscess. The liver abscess in our patient surrounded a branch of the hepatic artery. The infection caused inflammation and weakening of the arterial wall. This damage led to the formation of an aneurysm, an abnormal bulge in the blood vessel, called “mycotic” because it is caused by infection. This is an extremely rare occurrence post TARE. As such, even physicians regularly treating HCC patients are not aware of this possible complication. Such a mycotic aneurysm can rupture with catastrophic consequences.

Percutaneous drainage and prolonged antibiotics are the standard treatment, which our patient received for his liver abscess. Unfortunately, despite this, a mycotic aneurysm formed, which subsequently ruptured, leading to a near-catastrophic event. The purpose of this paper is to alert physicians to this unusual complication from TARE, such that prompt diagnosis and intervention can ensue.

## Introduction

Hepatocellular carcinoma (HCC) is the sixth most common cancer globally and the third leading cause of cancer-related deaths [1]. Over 30% of HCC in developed countries presents as an intermediate stage [2], which, according to guidelines [3], is typically treated with local regional transarterial therapy [4]. Since transarterial embolization was first reported in 1974, various modalities of treatment have evolved, initially with transarterial chemoembolization (TACE) [5], later with drug-eluting beads (DEB-TACE) [6], and more recently with transarterial radioembolization (TARE) [7]. A meta-analysis [8] found that TARE may offer advantages to TACE, including longer time to progression and fewer side effects. Other studies [9] have also shown that TARE can yield higher treatment response rates and prolonged survival. TARE has gained significant traction as the primary transarterial therapy modality of choice for HCC [10].

TARE is a form of selective internal radiation therapy (SIRT) that delivers radioactively labeled microspheres (commonly Yttrium-90 (Y-90)) directly into the arterial supply of a tumor, often through the hepatic artery. The microspheres lodge within the tumor's vasculature, delivering localized radiation while sparing surrounding healthy tissue. This minimally invasive procedure is preferred in patients who are not candidates for surgical resection or those with unresectable tumors [7].

While TARE has proven to be an effective palliative treatment option, it carries a risk of complications [11], particularly in patients with underlying vascular abnormalities or compromised immune systems. One of the most serious, but fortunately rare, complications is the development of liver abscess in the treated area [12]. Here, we present an unusual ramification resulting from a post-TARE liver abscess bleeding from a mycotic aneurysm arising in the liver abscess post TARE, which, to our knowledge, has not been previously described. This is a potentially life-threatening event that requires prompt recognition and intervention.

## Case presentation

**Figure 1:**
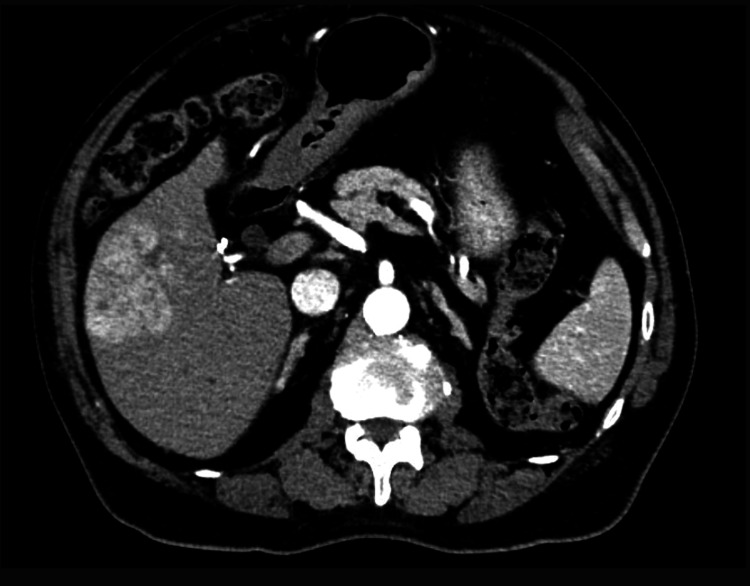
A CT scan of the abdomen showing right liver hepatocellular carcinoma at initial diagnosis

**Figure 2:**
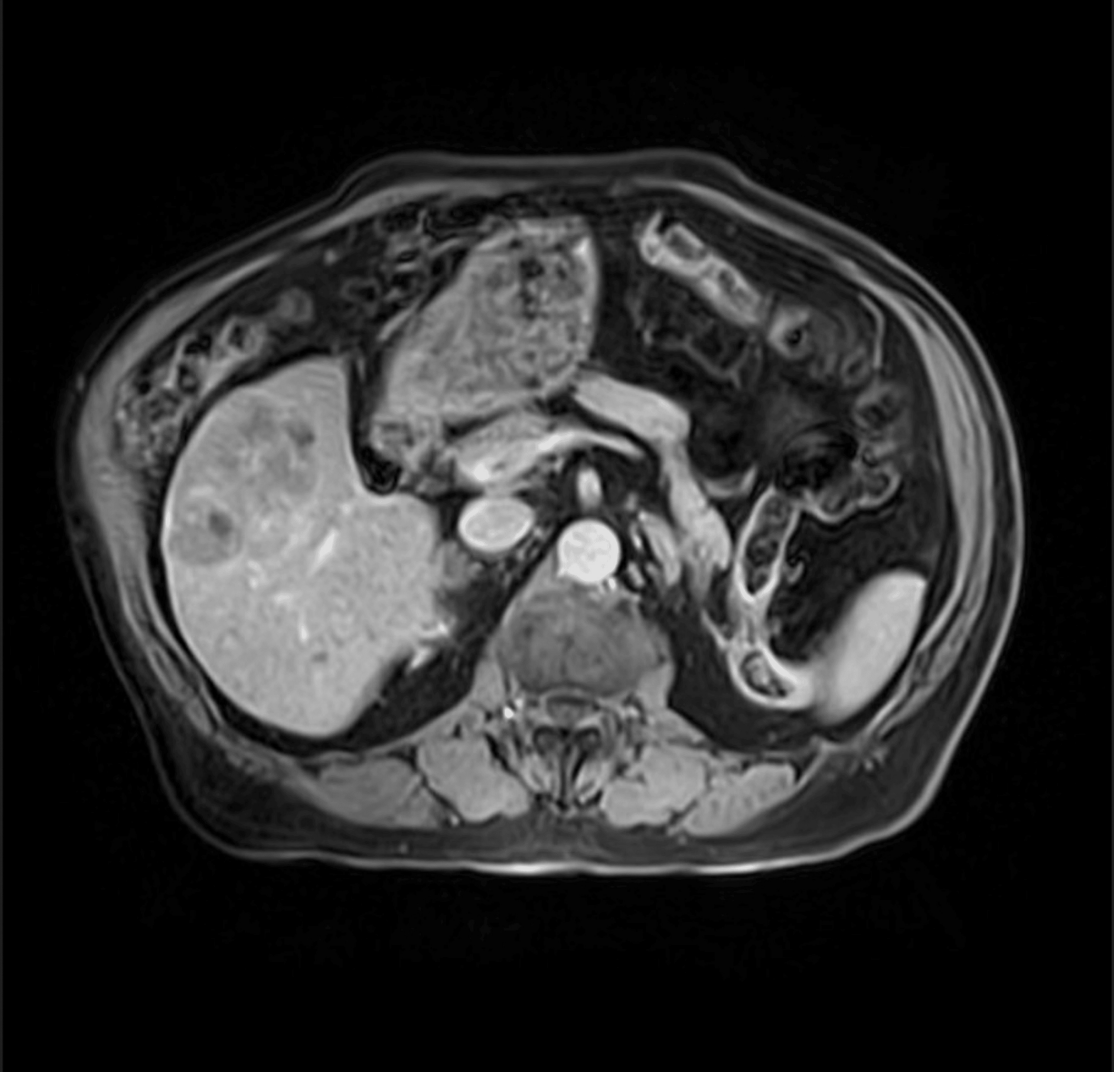
An MRI of the abdomen showing primary hepatocellular carcinoma with no intra-abdominal metastasis

**Figure 3:**
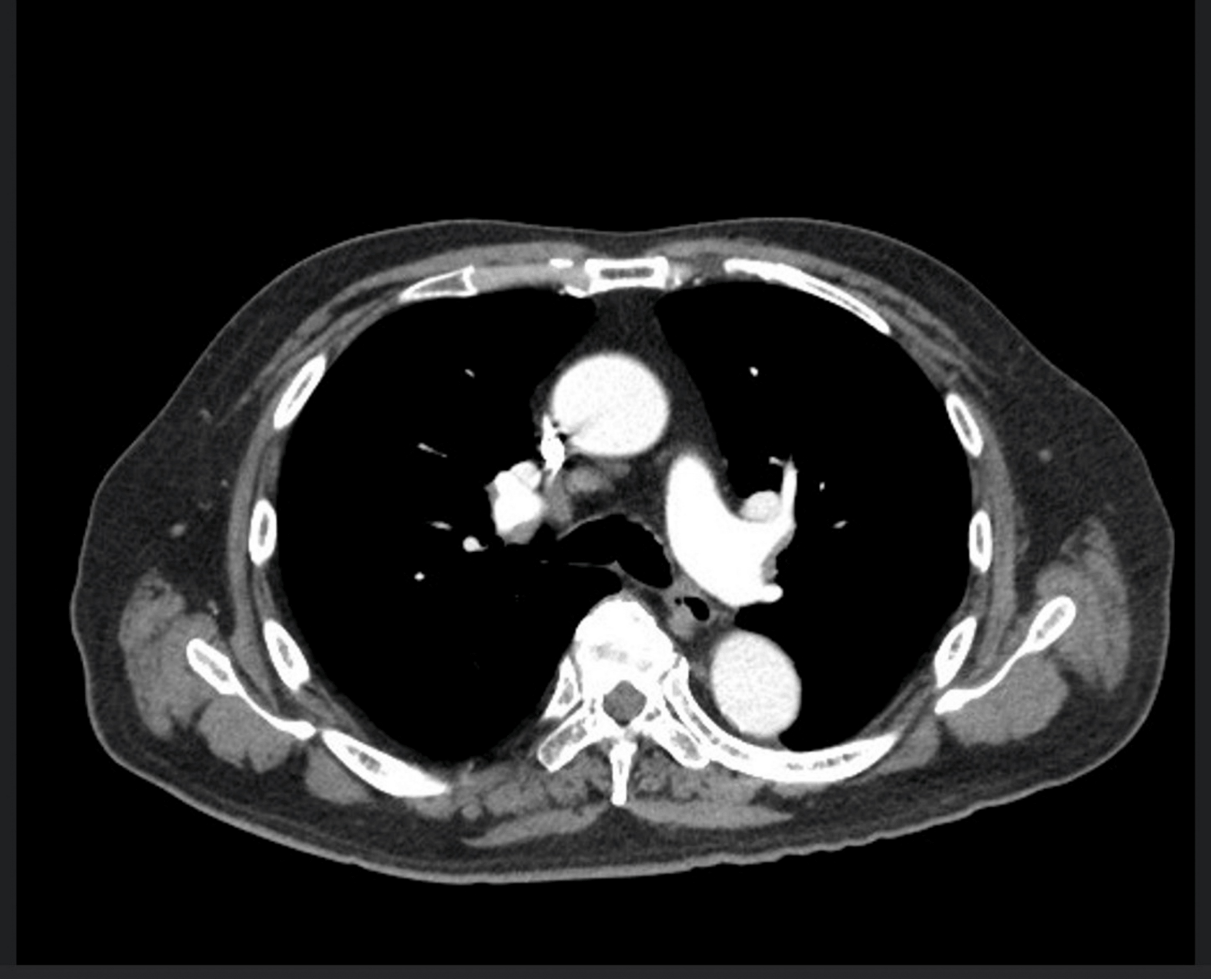
A CT scan of the chest showing no pulmonary metastasis

**Figure 4:**
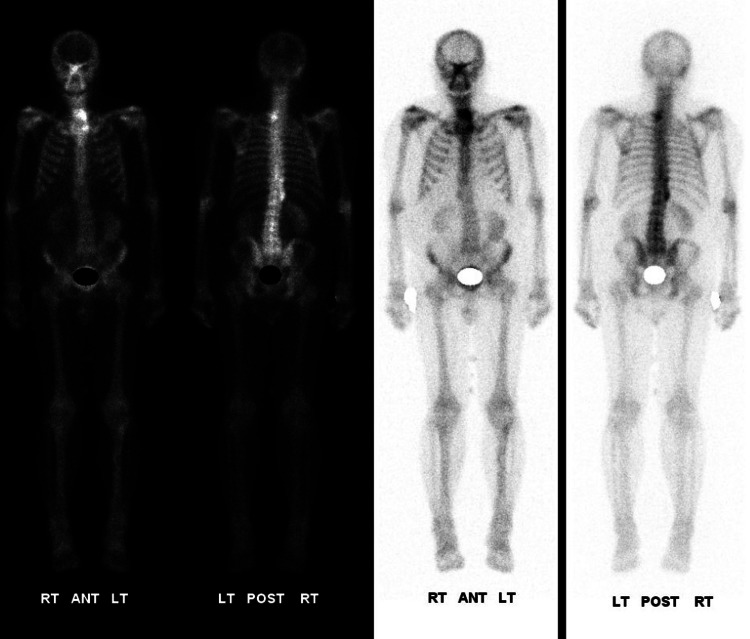
Bone scan showing no signs of metastasis

A 76-year-old male patient with adult-onset diabetes mellitus, atrial fibrillation, hypertension, and nonalcoholic steatohepatitis (NASH) presented in January 2024 with an incidentally discovered liver mass in segment 6, measuring 5.5 × 6.8 × 5.5 cm (Figure 1). MRI confirmed this lesion with no obvious intra-abdominal spread (Figure 2). The CT of the chest and bone scans were normal (Figures 3, 4).

**Figure 5:**
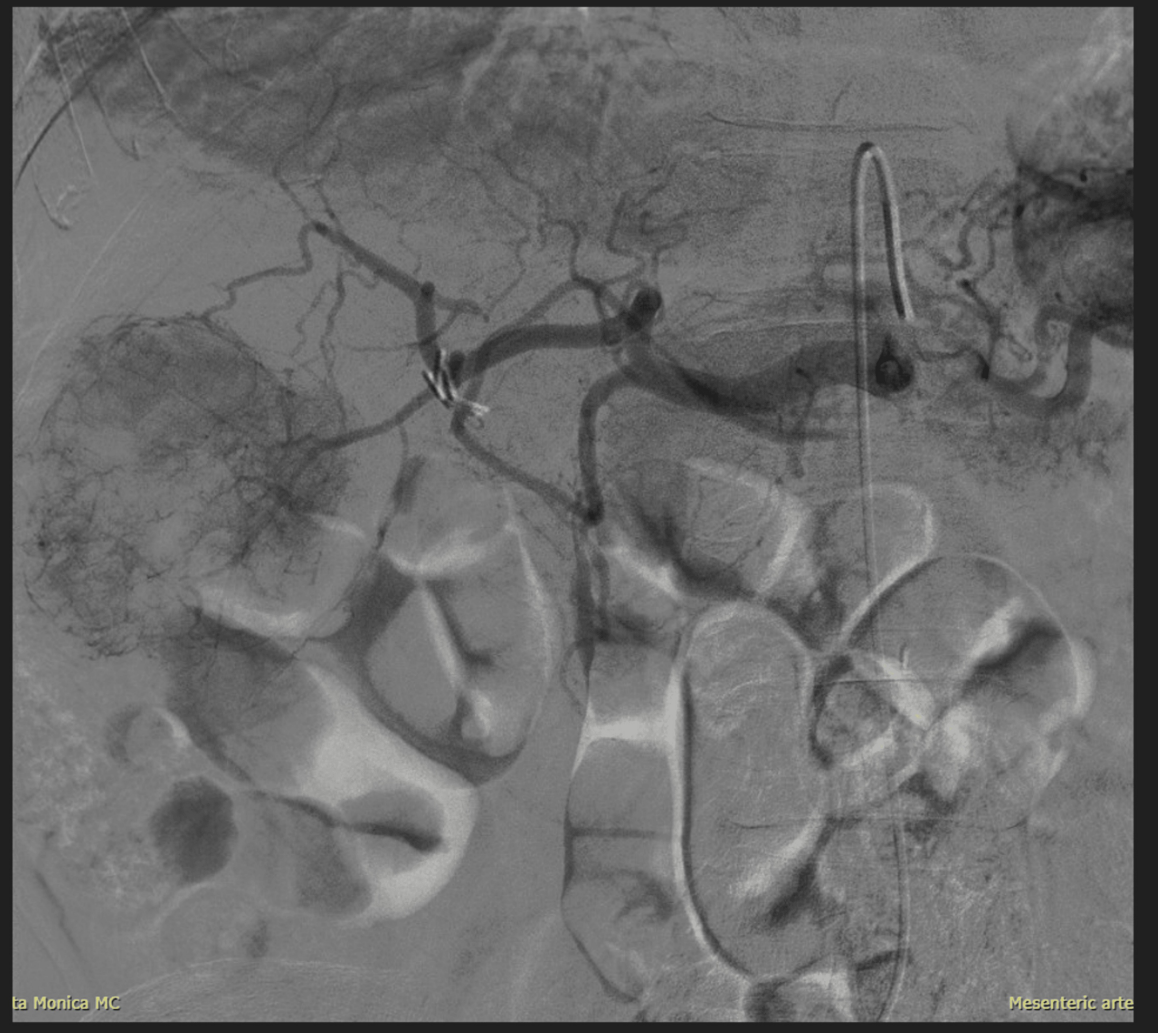
Yttrium-90 radioembolization of the tumor; tumor blush seen post injection

**Figure 6:**
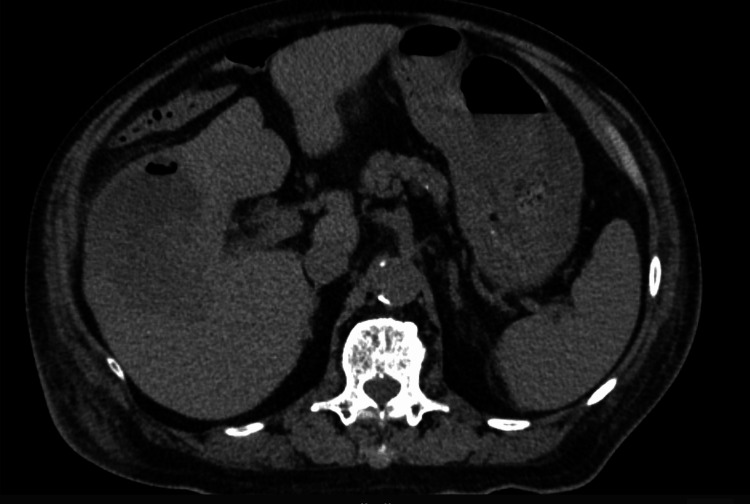
Liver abscess with air–fluid level following transarterial radioembolization

A US liver biopsy revealed well-differentiated HCC. Alpha-fetoprotein (AFP), carbohydrate antigen 19-9 (CA19-9), and hepatitis serologies were all within normal limits. The patient was taken to the operating room in February 2024 for liver surgery at the University of California, Los Angeles (UCLA) Hematology Oncology, Alhambra. Intraoperatively, the tumor was thought to be unresectable due to its volume and evidence of liver cirrhosis. The patient was subsequently seen by interventional radiology and underwent Y-90 radioembolization in March 2024 (Figure 5). Unfortunately, two to three weeks post TARE, the patient developed fever and sepsis with blood cultures positive for *Escherichia coli* (*E. coli*). A CT scan initially showed post-TARE changes in the liver. The patient was treated with four weeks of IV antibiotics, third-generation cephalosporin. Despite this, once antibiotics were stopped, he again developed recurrent* E. coli *bacteremia. A CT and MRI of the abdomen in June 2024 showed post-radioembolization changes with a large lobular fluid- and gas-containing collection within the right hepatic lobe treatment zone, consistent with an abscess (Figure 6). A percutaneous drainage tube was inserted along with continued antibiotic therapy.

**Figure 7:**
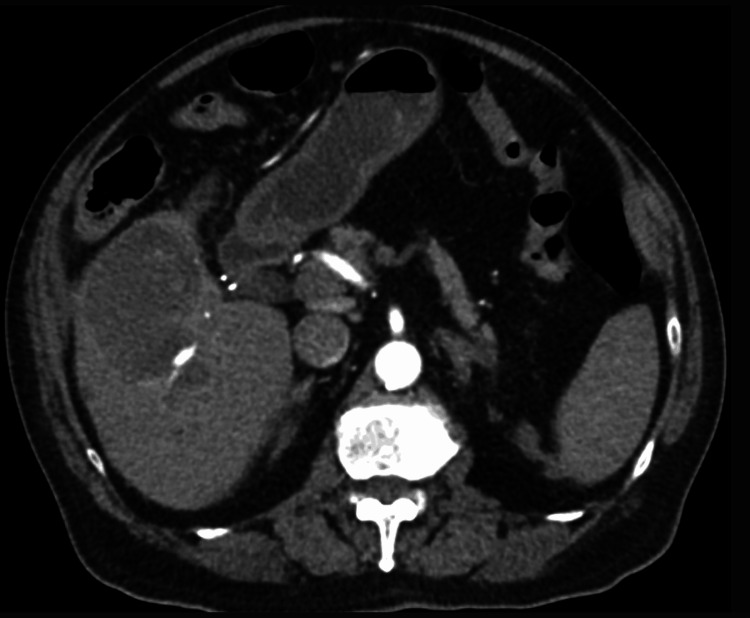
A CT scan of the abdomen showing a mycotic aneurysm in the posterior branch of the right hepatic artery within a liver abscess.

**Figure 8:**
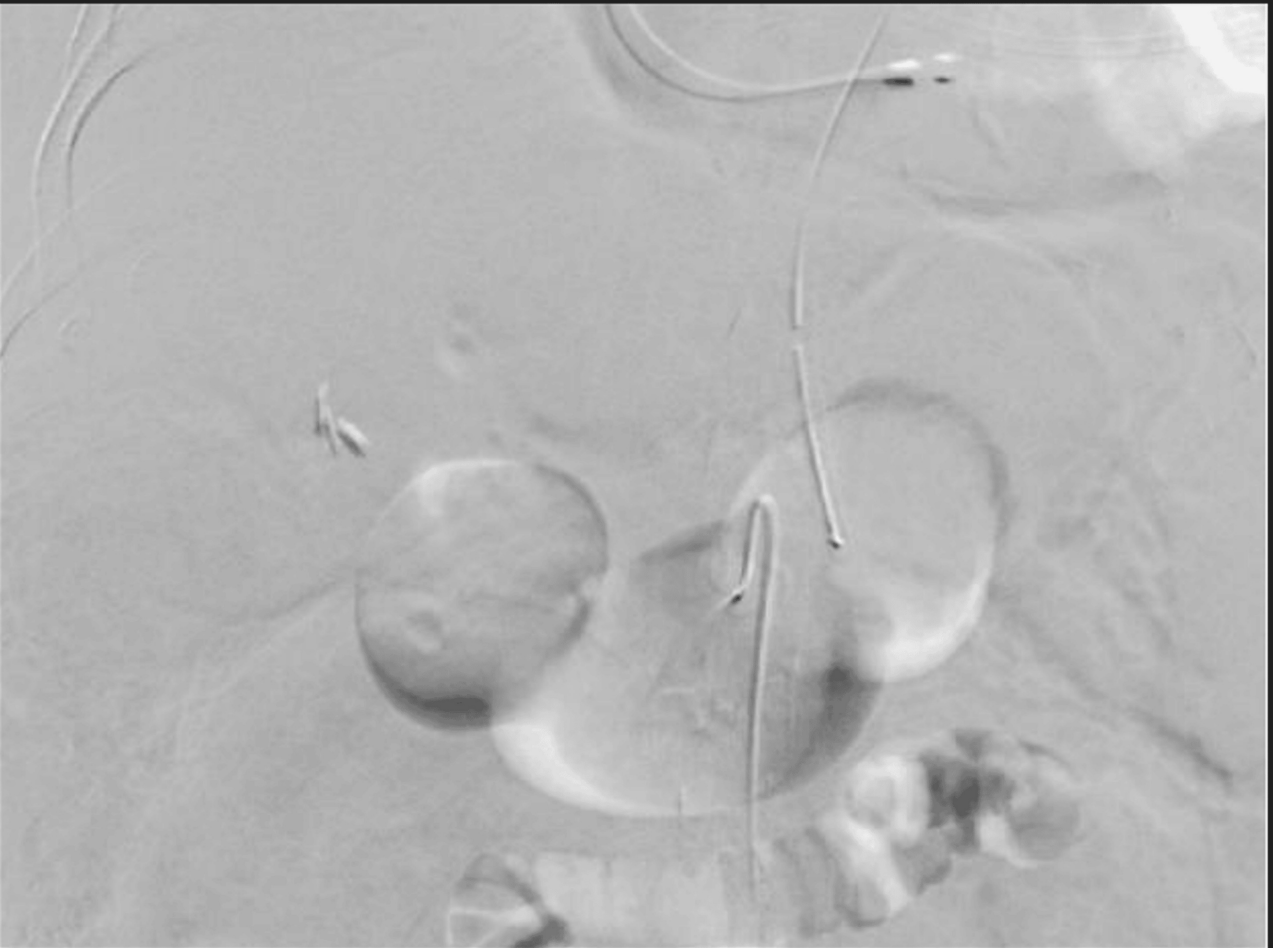
Selective coil embolization of the aneurysmal branch during a bleeding episode.

The patient was followed up with serial CT scans every one to two months, with a repeat scan in September 2024 showing a decrease in the size of the rim-enhancing right hepatic lobe treated zone, without washout, and Liver Imaging Reporting and Data System (LI-RADS) treatment response equivocal, but also noted an interval increase in the size of the dilated posterior right hepatic branch, measuring up to 1.3 cm, a finding suggestive of mycotic aneurysm (Figure 7). Interventional radiology was consulted. But unfortunately, prior to any procedure, the patient acutely bled from the tumor area, rupturing into the retroperitoneum, with severe hypotension resulting in multiorgan failure. The patient was intubated and resuscitated in the ICU and underwent emergency embolization of the bleeding posterior right hepatic artery aneurysm (Figure 8). Fortunately, the bleeding resolved, the patient recovered, and he was subsequently discharged.

The last clinic follow-up was in April 2025; the patient was alive and well. He continues on the prophylactic antibiotic, Levaquin.

## Discussion

HCC is the third leading cause of cancer-related deaths in the world and one of the fastest-growing causes of cancer death in the United States [1]. Patients typically present with locally advanced disease in a compromised liver, making it difficult to treat [2]. In unresectable cases, local regional therapy is normally recommended, as opposed to systemic treatment [3, 4]. The choice of local regional therapies includes local thermal ablation, TACE, TARE, hepatic artery infusional chemotherapy (HAIC), and stereotactic body radiation (SBRT) [5, 6, 7]. Both thermal ablation and SBRT work better for smaller tumors, less than 3 cm. HAIC is limited to specialized centers with technical expertise. TACE and TARE are the primary options for this patient [5, 6]. Recent meta-analyses comparing TACE versus TARE suggest TARE provides significantly longer time to progression compared with TACE with less toxicity. It also entails fewer treatments but at a higher cost [8].

TARE is a minimally invasive procedure used primarily for the treatment of liver cancer, particularly HCC [5]. During TARE, tiny radioactive beads are delivered into the blood vessels feeding the tumor. These beads emit radiation, which directly targets and destroys the tumor while minimizing damage to surrounding healthy tissue [7].

Though TARE is effective in treating liver cancer, it carries potential side effects. These include systemic side effects such as postembolization syndrome (PES) and lymphopenia, as well as hepatobiliary complications such as radioembolization-induced liver disease, liver infarction, and abscess. Also, TARE-specific complications such as radiation cholecystitis, pneumonitis, and gastrointestinal injury can arise [11].

A hepatic abscess is a rare but serious complication of Y90 TARE; few cases have been reported in the literature [12, 13]. Most reports are related to complications of TACE or DEB-TACE. In these patients, the reported incidence is 0.3%-5% [14, 15]. Patients with prior biliary intervention resulting in sphincter of Oddi compromise have an increased risk, likely related to biliary colonization and post-embolic biliary ischemia [16].

Patients with liver abscesses typically present with fever, pain, nausea, and vomiting, usually two to four weeks after treatment. Clinically distinguished from PES, which occurs one to two days post embolization, with similar symptoms. CT scans are necessary for prompt diagnosis. Features on the CT scan suggestive of an abscess include fluid, ring enhancement, and air. WBC count is typically elevated, and blood cultures are positive in over 50% of cases [17]. Prompt treatment with systemic antibiotics and drainage is necessary due to the risk of sepsis, as seen in our patient.

Mycotic aneurysms [18] are rare in their frequency but can be life-threatening with a high incidence of arterial rupture. A mycotic aneurysm normally arises from bacterial invasion into the arterial wall through hematogenous spread in immunocompromised patients with diabetes or liver cirrhosis. In patients post TARE, direct vessel wall injury from catheter manipulation or embolic particles can damage the endothelium. Radiation can cause local tissue injury, necrosis, and weakened vascular walls. Bacteria gain access (from a concurrent infection or bacteremia), and they can seed the weakened arterial wall. Also, underlying immunosuppression from diabetes and cirrhosis enhances predisposition [19].

The mycotic aneurysm found in the posterior branch of the right hepatic artery of our patient coursed through the post-TARE liver abscess. Causally, TARE-related vessel wall injury and subsequent bacterial seeding, both direct and hematogenous, related to the post-embolization liver abscess, in this older patient with baseline cirrhosis and diabetes, was the pathophysiology of this extremely rare complication.

## Conclusions

While TARE is an effective treatment for liver tumors, it carries a small but significant risk of complications. Liver abscess post TARE is rare compared to TACE, with only a series of case reports in the literature. To the best of our knowledge, a mycotic aneurysm related to a liver abscess post TARE, complicated by bleeding, has never been reported. In this case report, we attempt to highlight the possibility of such a rare vascular complication from TARE. Early identification, appropriate antimicrobial therapy, and targeted vascular interventions can significantly change patient outcomes. As TARE continues to gain popularity, clinicians must be vigilant in recognizing the signs of unusual vascular complications to ensure the best possible care for patients undergoing this procedure.
